# Food packaging permeability and composition dataset dedicated to text-mining^☆^

**DOI:** 10.1016/j.dib.2021.107135

**Published:** 2021-05-13

**Authors:** Martin Lentschat, Patrice Buche, Juliette Dibie-Barthelemy, Luc Menut, Mathieu Roche

**Affiliations:** a IATE, Univ Montpellier, INRAE, Institut Agro, 2 place Pierre Viala, Montpellier 34060, France; b TETIS, Univ. Montpellier, AgroParisTech, CIRAD, CNRS, INRAE, 500 Rue Jean François Breton, Montpellier 34090, France; c Univ. Paris-Saclay, INRAE, AgroParisTech, UMR MIA-Paris, 16, rue Claude Bernard, Paris 75231 CEDEX 05, France; d CIRAD, UMR TETIS, Montpellier F-34398, France

**Keywords:** Natural language processing, Food packaging, Ontology, Permeability, Quantity, Component

## Abstract

This dataset is composed of symbolic and quantitative entities concerning food packaging composition and gas permeability. It was created from 50 scientific articles in English registered in html format from several international journals on the ScienceDirect website. The files were annotated independently by three experts on a WebAnno server. The aim of the annotation task was to recognize all entities related to packaging permeability measures and packaging composition. This annotation task is driven by an Ontological and Terminological Resource (OTR). An annotation guideline was designed in a collective and iterative approach involving the annotators. This dataset can be used to train or evaluate natural language processing (NLP) approaches in experimental fields, such as specialized entity recognition (e.g. terms and variations, units of measure, complex numerical values) or sentence level binary relation (e.g. value to unit, term to acronym).

## Specifications Table

**Table:** 

Subject	Data Science: Data Mining and Statistical Analysis
Specific subject area	Food packaging permeability and component
Type of data	Table
How data were acquired	Data are the result of a manual annotation by three persons on a WebAnno 3.5.5 server [1]. The text of 50 papers from several journals were manually extracted from ScienceDirect and prepossessed. Pre-processing programs are available along with the dataset. Annotation was performed accordingly to annotation guidelines available within this dataset^1^.
	Only the annotated texts corresponding to short citations are available with their respective features, no full-text data are provided due to copyright observance.
Data format	Raw
Parameters for data collection	TRANSMAT [2], [3], [4] Ontological and Terminological Resource (OTR) has been used for data annotation. Articles which have been annotated were selected within the field of the OTR to answer specific Competency Questions (CQs) [5].
	CQ1: Which are the constituents of food packagings and associated quantities in the packaging composition?
	CQ2: Which are the O2/CO2/H2O permeability values and units associated with the different food packagings studied in the article?
	CQ3: Which are the controlled parameter values and units associated with O2/CO2/H2O permeability measurements?
Description of data collection	The dataset is constituted of four table files: one for each annotator and one for the Gold Standard built from the consensus between annotations. The table files describe each data through a set of features.
Data source location	The data are hosted on the CIRAD DATAVERSE. The data were manually collected within the UMR TETIS, TETIS, Univ. Montpellier, AgroParisTech, CIRAD, CNRS, INRAE, Montpellier, France and manually annotated on a WebAnno server hosted within the UMR IATE, Univ. Montpellier, INRAE, Institut Agro, Montpellier, France.
Data accessibility	Repository name: TRANSMAT Gold Standard Data identification number: https://doi.org/10.18167/DVN1/U7HK8J Direct URL to data: https://dataverse.cirad.fr/dataset.xhtml?persistentId=doi:10.18167/DVN1/U7HK8J
Related research article	M. Lentschat, P. Buche, J. Dibie-Barthelemy, M. Roche, Scipure: a new representation of textual data for entity identification from scientific publications, in: Proceedings of the 10th International Conference on Web Intelligence,Mining and Semantics, 2020, pp. 220-226.

1 https://dataverse.cirad.fr/dataset.xhtml?persistentId=doi:10.18167/DVN1/U7HK8J

## Value of the Data

• This dataset contributes to the available resources for natural language processing (NLP) on specialized domains, more precisely in new generation and bio-sourced food packaging field.
• This dataset is useful for computer scientists in NLP and data mining tasks.
• This dataset can be employed for evaluation or training on various tasks: specialized entity recognition (e.g. terminological variations, units of measure, complex numerical values); binary relation identification at sentence level (e.g. value to unit, term to acronym).
• The dataset is scalable as all processing codes are provided. It can be easily replicated and extended with additional documents.
• The annotators have identified a large variety of entities (e.g. packaging, temperature) of two categories (i.e. symbolic and quantitative). These entities are relevant to permeability and composition relations of food packaging.

## 1 Data Description

The dataset [6] is primarily constituted of four data-files (.csv). One annotation guide (.pdf) details the instruction to annotators and the choices made. An archive (.targz) contains necessary python codes to reproduce the transformation of the documents needed for the annotation. The four data files are distributed as follow: one for each of the annotators (goldenTRANSMATannotator1, goldenTRANSMATannotator2, goldenTRANSMATannotator3) and one aggregating the work of all annotators (goldenTRANSMATall). Each file organizes data in a table (an example of a data row is given in Table 1). The data are described thought a set of features:

**Doc** the article title from which the data was annotated;
**DOI** the Digital Object Identifier of each article;
**Target** the generic concept represented by the data in the TRANSMAT OTR;
**Type** the ontology concept category, symbolic, quantitative or adimensional;
**Original_Value** the text annotated constituting the data: a list of annotated tokens for symbolic data, two lists of annotated tokens for quantitative data (a list of numerical values and a list of measurement units);
**Attached_Value** the list of annotated tokens to disambiguate a measure unit when necessary for quantitative data. None for symbolic data.
**Annotator** the annotator id.

**Table 1:** Example of a row in the goldenTRANSMATall.csv file.

Features	Values
Document	Barrier and surface properties of chitosan-coated greaseproof paper
DOI	https://doi.org/10.1016/j.carbpol.2006.02.005
Target	permeability
Original_Value	*([’3400’], [’cm’, ’^’, ’3’, ’mm’, ’/’, ’(’, ’m’, ’^’, ’2’, ’atm’, ’day’, ’)’])*
Attached_Value	*[’carbon’, ’dioxide’]*
Type	QUANTITY
Annotator	1

These data files were used as a Gold Standard in a study on experimental data extraction and relevance [7].

The annotation guideline (guideAnnoTRANSMAT_english.pdf) presents the WebAnno interface and the instructions to annotators. These instructions and the choices made are summarized in the next section.

The annotation framework defines several tags to annotate the texts. Symbolic concepts are annotated with three tags: *Packaging* is used to annotate a full packaging name, *Packaging_Component* is used to annotate a specific component of a packaging and, *Method* is used to annotate the name of the method used for permeability measurement. Quantitative concepts are annotated with six tags that cover terms describing experimental controlled and measurement parameters: *Temperature, Thickness, Relative_Humidity, Relative_Humidity_Difference, Partial_Pressure_Difference* and *CO2/H2O/O2_Permeability*. Their numerical values are annotated with *numeric_Value* tag and measurement units with *measure_Unit* tag. Number and distribution of annotated information in the corpus are given in Table 2.

**Table 2:** Distribution of the data in the Dataset files.

	annotator 1	annotator 2	annotator 3	all
Target	50 documents	first 5 documents	last 5 documents	50 documents
SYMBOLIC	988	127	42	1050
packaging	431	60	30	476
method	43	7	5	46
impact_factor_component	514	60	7	528
QUANTITATIVE	686	30	81	722
component_qty_value	365	16	13	379
permeability	150	6	42	165
relative_humidity	58	3	9	61
temperature	54	4	9	56
thickness	44	1	5	45
partial_pressure_difference	15	0	3	16
TOTAL	1676	157	123	1772

## 2 Experimental Design, Materials and Methods

The dataset was obtained from 50 articles gathered from different journals on the website ScienceDirect in html format.

TRANSMAT [2], [3], [4] Ontological and Terminological Resource (OTR) represents concepts and their relations in the food packaging domain. The OTR is structured in a core ontology and a domain ontology. The up-core ontology includes a representation of the structure in n-Ary relations and their arguments. The down-core ontology contains the generic concepts specific to the experimental fields, such as quantitative or symbolic concepts and measurement units. The domain ontology contains concepts related to the specific field of interest, i.e. food packaging permeability. Each symbolic or quantitative concept is associated with a terminological component in the form of *labels* (*preferred* or *alternative*). Quantitative concepts are also associated with measurement unit concepts. Each measurement unit is associated with a set of labels. The OTR defines the concept of interest of each relation and their associated vocabularies. The measurement units and *labels* of the concepts involved in the n-Ary relations of interest define the tokens forming the entities in the documents.

The documents were transformed from their html format into text format (codes for this task are available within the dataset). The purpose was to obtained files suitable for annotation on the WebAnno server. The files have been cleaned of all html related information to retain only the textual (i.e. text body) and structural (i.e. section and sub-section names) information. In the figures (e.g. graphs, photos, etc.) the caption and title were kept and included in the text body, with the hyperlink to the figure if available. The characters encoding was also normalized into UTF-8. The text treatment and segmentation applied to the sentences were realized using the Stanza [8] library. Tables were parsed and re-structured in a format suitable for WebAnno. This involves the re-structuring of the table content and recognition of the caption and table title. All articles related information such as copyright, authors list or references were discarded.

**Fig. 1:**
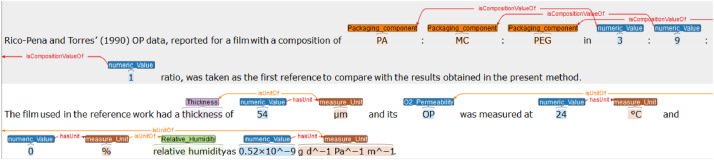
An excerpt of the WebAnno annotation process.

The annotation was made on a WebAnno server deployed on a docker server in the JRU IATE (Montpellier, France). An excerpt of the annotation task is presented in Fig. 1.

Two n-Ary relations of interest were selected in the OTR to be annotated: the permeability relations (oxygen, carbon dioxide and water permeability) and food packaging composition (impact factor component). Only entities related to these relations were annotated (e.g. a temperature used as control parameter for a permeability measure is annotated, a temperature related to storage condition is not annotated). The annotation task was first made on a sub-corpus of 10 documents by the first annotator who conceived a preliminary annotation framework. This task consists in identifying all information that constitute an argument for the two n-Ary relations of interest. Each of these relations includes different arguments represented in the texts by symbolic and quantitative information. The final version of the annotation framework was obtained after an iterative process including all annotators. Let us notice that the WebAnno interface is not designed to annotate data in tables. In order to ease the annotation task, information with exactly similar character sequences present at different locations in the documents was not annotated, unless necessary (e.g. if the duplicate entity is connected to another entity of interest). This choice was made because many duplicates were present in the documents, but the annotation of all occurrences does not generate useful information for the task at hand.

Oriented binary relations are also used to link specific tags. Two binary relations exist for quantitative entities: *hasUnit* links a *numeric_Value* tag to a *measure_Unit* one, *isUnitOf* links a *measure_Unit* tag to its experimental parameters tag (e.g. *”*${}^{\circ }C$*”* to *”temperature”*). Moreover, *isCompositionValueOf* links a numerical value or a measure unit to a *Packaging_Component* tag (a packaging composition value may be adimentional).

The annotation was made by three annotators. The first one annotated all the corpus and the others two annotated five different documents each (respectively the first five and last five documents in alphabetical order). A summary of the annotation is given in Table 2. This allows the computation of agreement scores: Two scores were computed on the overlapping documents, between annotators 1 and 2 and between annotators 1 and 3. The integrated WebAnno agreement computation tool DKPro Statistics [9] measured an average Cohen’s Kappa of $\kappa_{C}=.98$. However, this agreement tool compares the tags with similar positions but not the tags assigned to same words present in different locations in the document.

The dataset is composed of the annotations extracted from WebAnno. The extraction program, available in the dataset, conserves only the textual entities and their annotated categories. Discarding all other textual content was made in order to observe the copyrights associated with the articles, not all paper being in Open Access. Note that the original DOIs and document names constitute one feature of the data.

The aggregation process of the annotations in an unique file to constitute the Gold Standard has to deal with the different tag choices made by the annotators. The annotation distributions presented in Table 2 show that the final Gold Standard is mainly constituted of annotations made by the annotator 1. Annotations of annotators 2 and 3 complete the Gold Standard. To address the differences between the tags assigned to the information, the first annotator was given the role of curator and decided the final annotation. The number and distribution of information in the final annotation is summarized in the last column of the Table 2.

## Ethics Statement

No conflict of interest exists in this submission. The authors declare that the work described in this paper is original and not under consideration for publication elsewhere, in whole or in part. Its publication is approved by all the authors listed.

## CRediT Author Statement

**Martin Lentschat:** Methodology, Software, Investigation, Resources, Data Curation, Writing - Original Draft, Visualization; **Patrice Buche:** Investigation, Validation, Writing - Review & Editing, Supervision; **Juliette Dibie-Barthelemy:** Writing - Review & Editing Luc Menut: Investigation, Validation; **Mathieu Roche:** Writing - Review & Editing.

## Declaration of Competing Interest

The authors declare that they have no financial or personal interests that could influence the work reported in this paper.
